# Nanoscale regulation of L-type calcium channels differentiates between ischemic and dilated cardiomyopathies.

**DOI:** 10.1016/j.ebiom.2020.102845

**Published:** 2020-06-21

**Authors:** Jose L. Sanchez-Alonso, Alexandra Loucks, Sophie Schobesberger, Ankie M. van Cromvoirt, Claire Poulet, Rasheda A. Chowdhury, Natalia Trayanova, Julia Gorelik

**Affiliations:** a Department of Cardiovascular Sciences, Imperial Centre for Translational and Experimental Medicine, National Heart and Lung Institute, Imperial College London, London W120NN, UK; b Department of Biomedical Engineering and Alliance for Cardiovascular Diagnostic and Treatment Innovation, Johns Hopkins University, Baltimore, MD 21218, USA

**Keywords:** Electrophysiology, Ion channels, Computational biology, Cardiomyopathy, Heart Failure, LTCC, L-type Ca^2+^ channels, HF, heart failure, TT, Transverse tubule (T-Tubule), LVAD, Left Ventricular Assistant Device, P_o_, open probability, EADs, Early afterdepolarizations, I_Ca,L_, L-type calcium current, ICM, Ischemic cardiomyopathy, DCM, Dilated cardiomyopathy, PKA, Protein kinase A, CaMKII, Calcium-calmodulin kinase II, SICM, Scanning ion conductance microscopy

## Abstract

**Background:**

Subcellular localization and function of L-type calcium channels (LTCCs) play an important role in regulating contraction of cardiomyocytes. Understanding how this is affected by the disruption of transverse tubules during heart failure could lead to new insights into the disease.

**Methods:**

Cardiomyocytes were isolated from healthy donor hearts, as well as from patients with cardiomyopathies and with left ventricular assist devices. Scanning ion conductance and confocal microscopy was used to study membrane structures in the cells. Super-resolution scanning patch-clamp was used to examine LTCC function in different microdomains. Computational modeling predicted the impact of these changes to arrhythmogenesis at the whole-heart level.

**Findings:**

We showed that loss of structural organization in failing myocytes leads to re-distribution of functional LTCCs from the T-tubules to the sarcolemma. In ischemic cardiomyopathy, the increased LTCC open probability in the T-tubules depends on the phosphorylation by protein kinase A, whereas in dilated cardiomyopathy, the increased LTCC opening probability in the sarcolemma results from enhanced phosphorylation by calcium-calmodulin kinase II. LVAD implantation corrected LTCCs pathophysiological activity, although it did not improve their distribution. Using computational modeling in a 3D anatomically-realistic human ventricular model, we showed how LTCC location and activity can trigger heart rhythm disorders of different severity.

**Interpretation:**

Our findings demonstrate that LTCC redistribution and function differentiate between disease aetiologies. The subcellular changes observed in specific microdomains could be the consequence of the action of distinct protein kinases.

**Funding:**

This work was supported by 10.13039/100000002NIH grant (ROI-HL 126802 to NT-JG) and British Heart Foundation (grant RG/17/13/33173 to JG, project grant PG/16/17/32069 to RAC). Funders had no role in study design, data collection, data analysis, interpretation, writing of the report

**Research in context**

**Evidence before this study**

Although cardiomyocyte L-type calcium channels, essentials for the contraction of the heart, have been the focus of studies of human heart failure in the last decades, no emphasis has been placed on their regional variation within the cell. The localization of these channels within cell membrane, has been found to be altered by the remodeling that cells undergo during the progression of heart failure.

**Added value of this study**

In this work, using a combination of microscopy and electrophysiological techniques, we study human cardiomyocytes from ischemic and dilated cardiomyopathy patients, and the impact that the left ventricular assistant devices have of them. Extensive remodeling of the cellular structures is stablish, which leads to redistribution of L-type calcium channels. Furthermore, the spatial location and function of L-type calcium channels is found to be different between ischemic and dilated cardiomyopathies. Changes that we found in each of the diseases are connected to a specific and different intracellular signaling pathway. Using computational simulations of the whole heart, we reveal the implications that these findings could have in heart rhythm dysfunction.

**Implications of all the available evidence**

Ischemic and dilated cardiomyopathies are two pathologies that are different in origin but share the same endpoint, which is the need for a heart transplant. This study found that despite the same end-stage time point, major differences exist at the sub-cellular level between aetiologies. These findings describe new targets specific to each disease, and this could open the door to new pharmacological treatments.

Alt-text: Unlabelled box

## 1 Introduction

The progression of structural heart diseases from an early form towards advanced heart failure (HF) is accompanied by an increased risk of arrhythmia and sudden cardiac death [1,2]. As preventing arrhythmia is a clinical goal of paramount significance and high urgency [3], a better understanding of arrhythmogenesis at the cellular level is a fundamental step towards achieving this goal.

Calcium signaling plays a critical role in the pathogenesis of heart failure. L-type calcium channels (LTCCs) are the main mediators of calcium influx into cardiac cells and are essential in determining the electrical and mechanical properties of cardiac muscle [4]. The regulation of this calcium influx depends on both the single channel activity and the localization of channels within the plasma membrane [5]. In fact, the specificity, reliability, and accuracy of autonomic modulation of the heart at the cellular level depend on tightly regulated spatiotemporal calcium signals restricted to precise microdomains. The role of L-type calcium current (I_Ca,L_) in the progression of HF remains, however, not well understood. Although Ca_V_1.2 channel density has been shown to be decreased on the surface of failing human cardiomyocytes [6], baseline whole-cell I_Ca,L_ has been widely reported to not be altered [7], [8], [9]. This suggests that LTCCs in HF may be hyperphosphorylated under basal conditions to compensate for channel loss. In fact, studies on single channel recordings by other authors [10] and us [11] have shown an increase in LTCC open probability (P_o_) in human HF, which could be linked to a higher phosphorylation of the channel.

In adult ventricular myocytes, a critical subpopulation of LTCCs, which participates in excitation-contraction coupling, is located in the specialized transverse tubules (T-tubules, TT) microdomains. These structures represent highly branched invaginations of the cardiomyocyte sarcolemma [12]. TT are highly specialized Ca^2+^-handling microdomains and their integrity underlies the normal contractile function of the human myocardium [13]. Several studies, including our previous investigations, have found that in HF, TT structures are progressively lost, with consequent changes in cell surface topography [14], [15], [16]. Functional changes in LTCC current have also been linked to TT loss. Bryant et al. showed that LTCC current density decreases at TT but increases at the sarcolemma surface in an HF rat model [17]. We obtained similar results regarding LTCC channel redistribution from TT to the surface membrane (the cell crest), both in a rat model of myocardial infarction and in patients with dilated cardiomyopathy. We previously demonstrated how this imbalance of LTCC at the cell membrane could lead to arrhythmia in the whole heart [11].

In the current study, conducted exclusively on control and failing human cardiomyocytes, we analysed and compared nanoscale LTCC distributions from healthy donor hearts, failing ischemic cardiomyopathy (ICM) hearts, and failing dilated cardiomyopathy (DCM) hearts, as well as from failing hearts after the implantation of left ventricular assist device (LVAD). We used a combination of electrophysiological and optical techniques and a multi-scale computational model to understand how changes at the cellular level affect the behavior of the whole organ and how different aetiologies may be distinguished.

## 2 Methods

### 2.1 Study approval

Experiments on isolated human cardiomyocytes were approved by Imperial College Institutional Review Board. Informed consent was taken from each patient. Samples from failing hearts of patients with end-stage heart failure were used with the approval from Brompton Harefield & NHLI Ethics Committee under Biobank REC approval reference 09/H0504/104+5. Samples from donor hearts unsuitable for transplantation were used with the approval of NHS BT with REC approval reference: 16/LO/1568.

### 2.2 Study population

Failing ventricular myocytes were prepared from hearts of patients with end-stage heart failure caused by ischemic or non-ischemic cardiomyopathies who were undergoing transplantation (*n* = 31 patients, average age 49.6 ± 1.78 years, 6 females and 25 males). Clinical characteristics of the heart failure patients used in this work can be found in Table 2. We used two population of ventricular samples as control for this study. The first one was from biopsies obtained from non-failing patients (*n* = 5, average age 69±2 years, two females and three males), with their consent during valve replacement procedure at Hammersmith Hospital, Imperial College London, London, UK. The second one was obtained from samples of left ventricles taken from donor hearts unsuitable for transplantation (*n* = 3, 47.1 ± 3.2, males). None of the donors had a history of hypertension, cardiothoracic disease, or diabetes. Two of them presented a history of smoking. The reasons for these hearts being rejected for transplantations were: degeneration on the organ care system prior to transplantation; heart size/logistics; and increased lactate and *K*+.

Although biopsies from Hammersmith patients were complicated by various factors, including age, early stage hypertrophy, atrial fibrillation, and coronary disease, they possessed normal left ventricle function (ejection fraction >60%). No differences in the parameters checked in this work were observed between these 2 populations of control cells, apart from the number of cells available after the isolation, which was drastically lower from the biopsy samples, due to the intrinsic small size of the sample.

### 2.3 Myocyte isolation

Ventricular cardiomyocytes from failing and donor hearts were isolated as previously described [11,14]. Due to cardiomyocyte heterogenicity throughout the myocardium, samples were taken from the same anatomical apical section of the posterior-lateral left ventricular free wall to avoid differences between the apex and the basal cells. ICM samples were taken from that areas that are not in the proximity of the scar or border zone. Ventricular cardiomyocytes were also isolated from biopsies from the left ventricle papillary muscles. In short, dissected samples were kept in ice-cold calcium free Krebs-Ringer saline solution (in g/L): 7.012 NaCl, 0.402 KCl, 1.332 MgSO[4], 0.55 Pyruvate, 3.603 Glucose, 2.502 Taurine, 2.383 HEPES, 1.286 Nitrilotriacetic Acid; pH = 6.96. Approximately 0.5 g of myocardial wall was taken, connective and adipose tissue was removed, and the cardiac muscle was cut with razor blades in small cubes of similar size (1–2 mm^3^). The cubes were transferred to fresh calcium free Krebs-Ringer solution at 37 °C and washed 3 times for 3 min each. After this, the tissue samples were incubated in 10 ml of Krebs-Ringer solution (in g/L): NaCl 7.012, KCl 0.402, MgSO4 1.332, Pyruvate 0.55, Glucose 3.603, Taurine 2.502, HEPES 2.383; pH = 7.4, adding 200 nM CaCl[2] and Proteinase type XXIV (0.36 mg/ml; Sigma-Aldrich) under mechanical agitation. After 25 min, the partially digested tissue was transferred to 10 ml of fresh Krebs-Ringer in which proteinase was substituted by collagenase type XIV (1 mg/ml Sigma-Aldrich) continuing at 37 °C under agitation. Every 10–15 min the tissue was transferred to a fresh solution while the remaining solution contained single isolated cardiomyocytes. Single rod-shape cardiomyocytes were visible under light microscopy after the first 10–15 min, with the biggest density of cells being obtained after another 10–15 min. Supernatants were centrifuged for 3 min at 600 rpm after each incubation step, and the pellets were re-suspended in 2–3 mL of Krebs-Ringer solution without enzymes. Cardiomyocytes were plated on dishes coated with laminin and left to stick to bottom for at least 45 min before experiments. Cardiomyocytes were used on the same day of isolation for an average time of 7 h, with a maximum time of 12 h.

End-stage heart failure was caused by ischemic (*n* = 10), dilated (*n* = 21), and myocarditis (*n* = 1) cardiomyopathy. Among these patients, 4 from the ischemic group, 8 from the dilated group, and the patient with myocarditis were implanted with LVAD. As control, 3 isolations were done in donor hearts not suitable for transplantation and 5 isolations were performed on left ventricular biopsies samples from papillary muscles. In total, 40 isolations were done for this study. Data extracted from 11 of these isolations has already been published in Circulation Research [11], corresponding exclusively to a part of the dilated cardiomyopathy group (excluding LVAD patients) and the biopsies samples from papillary muscles.

### 2.4 Size, T-tubule and surface characterization of ventricular cardiomyocytes

Ventricular cardiomyocytes from all groups were used to study the T-tubule network, the surface morphology, and the size of the cells. A Zeiss LSM-780 inverted confocal microcopy was used to visualize the subcellular T-Tubule structure. Cells were stained with Di-8-ANEPPS [14] and taken with a 63x magnification objective. T-Tubule density was calculated using Image J software. Only the area inside the cell was selected, automatically thresholded and binarized. The ratio of black pixels to white pixels determined the density.

The TT regularity was calculated from 40 × 5 micros rectangles inside the cell (avoiding the cell nuclei) using a single dimension Fourier transformation from a custom-written macro for Matlab (The MathWorks, Inc., Natick, MA, USA) as described before [61].

Z-groove index was characterized by scanning ion conductance microscopy (SICM) which produced a 3D topographical map of the surface [62].

To maximize the number of measures, cell length and width were obtained from bright field and confocal images. Only cells with clear cross-stations and rod-shaped were selected.

### 2.5 Super-resolution scanning patch-clamp with pipette clipping modification

Cell-attached patch-clamp recordings of single L-type calcium channels were obtained and classified based of the specific location of the pipette: on T-Tubule or crest microdomain. T-Tubules are defined in the SICM images as deep rounded or semi-rounded invaginations, connected by less deeper striations which define the Z-grooves. Crest areas are the spaces between the Z-groove lines, occupying most of the topographical surface in some images from heart failure cardiomyocytes. The use of SICM with pipette clipping modification [63] for the study of single channels has been successfully applied in several publications [11,12,64]. Controlled widening of the scanning nano-pipette tip has been previously described in detail [11].

Experiments were performed at room temperature using the following solutions; external solution containing in (mmol/L): 120 K-gluconate, 25 KCl, 2 MgCl2, 1 CaCl2, 2 EGTA, 10 Glucose, 10 HEPES, pH 7.4 with NaOH, ∼290 mOsm; internal recording solution containing in (mmol/L): 90 BaCl2, 10 HEPES, 10 Sucrose, pH 7.4 with TEA-OH, ∼250 mOsm. For the experiments with the inhibitors H-89 (10 µM) and KN-93 (5 µM), cells were incubated with the blocker for a minimum 5 min before the first cell was patched, and each dish was used for a maximum of one hour. The pipette used for cell attached recordings after clipping had an average resistance of 27.78 ± 0.26 MΩ. Currents were recorded using Axopatch 200A amplifier (Axon Instruments, Foster City, CA, USA), controlled and monitored using pClamp software version 10 (Axon Instruments). Liquid junction potential was calculated as −16.7 mV and corrected for all the data shown in this work. Single LTCCs were identified and characterized by their voltage dependent properties. After performing a seal, the holding membrane potential was held at −96.7 mV. A protocol of incremental step of 10 mV with pulses from −36.7 to +23.3 mV was applied a minimum of 3 times. A current-voltage (I-V) relationship was generated form this data, when a channel was present. For the study of the Po the −6.7 mV step voltage was choose and a protocol of 50 consecutives pulses at this voltage was applied a minimum of 3 times. Single channels were sampled at 10 kHz and filtered at 2 kHz (−3 dB, 8-pole Bessel). Analysis was performed using Clampfit version 10.2 All the sweeps were checked for the presence of LTCCs.

Occurrence of LTCCs was calculated as the percentage of recording showing activity versus the total number of recordings. Channel density was calculated for each group, as the relation between the total area sealed (all recordings including the ones without presence of LTCCs) and the total number of channels. The area sealed under the pipette for each recording was estimated from the resistance of the pipette for each experiment as described by Novak et al. [63].

The Po was averaged from a minimum of 20 sweeps at −6.7 mV for each cell. Each cell was recorded only once. The total number of channels in the recording was input into pClamp software to calculate the single Po of one single channel. To estimate the number of channels per seal a carefully examination of all the recordings per cell was done. The level of the spikes determined the number of channels present in the seal. When only a single level of spikes was observed that seal was considered to contain only one channel.

The conductance was determined by plotting the average amplitude of all the openings in the recording against the test potential for every single experiment. The slopes from the linear relationships of these I/V plots were calculated as the conductance for that single channel.

### 2.6 Single cell modeling methodology

The O'Hara-Rudy ionic model [65] was used to represent the electrophysiology of healthy human ventricular cardiomyocytes. A number of changes in the formulation of the human LTCC current was introduced to represent the subcellular remodeling observed in the different aetiologies of cardiomyopathy. Myocytes from dilated human hearts were modeled by incorporating the changes in the ionic model described in our previous work [11]. To model cells from ICM, modifications were incorporated based on the experimental data acquired here, namely TT density, regularity, and channel occurrence. To model the loss of TTs observed experimentally in ICM, the parameter TTD encoding for TT integrity was introduced, with a value of 0.65 calculated using the equation:

$$\text{TTD}=(\frac{\text{TT density in ICM}}{\text{TT density in control}}+\frac{\text{Z groove ratio in ICM}}{\text{Z groove ratio in control}})\cdot \frac{1}{2}$$

Then, two new parameters encoding for the distribution of LTCCs between the TT and the Crest domains were introduced. Thus, the ratio between the channels located in the TT domain to the channels located in the Crest was found to be 0.8 using the equation:

$$\frac{⧣\text{channels in TT}}{⧣\text{channels in crest}}=\frac{\text{Percentage of recordings with LTCCs on the TT}}{\text{Percentage of recordings with LTCCs on the Crest}}$$

Therefore, a fraction of approximately 0.45 of LTCCs within a cell were located on the TT membrane, while the remaining 0.55 were relocated to the Crest.

As the experimental evidence from human myocytes indicated that remodeling has no effect on the peak of the LTCC current [65], the whole cell LTCC current was adjusted to match that of control in terms of magnitude, by multiplying it with a correction factor.

Following the experimental evidence here, PKA was incorporated into the ICM cardiomyocyte model and allowed to interact with the LTCCs present in the TT domain. Similar to the O'Hara-Rudy human ventricular model combined with the electrophysiological module of the Heijman beta adrenergic model [66] described previously [67], when PKA phosphorylated a LTCC, the current through that channel was increased by a factor of 2.5 and the gating parameters were updated.. Similar to the Sanchez et al. study [11], a stochastic Markov formulation was used to model the single channel behavior and to determine the appropriate fraction of channels which would undergo PKA phosphorylation in ICM TTs in order to match the experimentally observed open probability value. The same 32 states from the Markov model described in our previous study were used to represent unphosphorylated channels. By introducing PKA phosphorylation, 32 additional equivalent states were created, all characterized by different gating kinetics as described in the O'Hara-Rudy model combined with the Heijman model [65,67]. By stochastically simulating the behavior of 1000 channels at a voltage of −6.7 mV, only a fraction of 0.7 LTCC channels needed to be phosphorylated in order to match the desired open probability. This is different from the DCM model, in which all channels in the crest were phosphorylated by CaMKII. After computing this fraction, the Markov model was reverted to the equivalent Hodgkin-Huxley formalism.

Apart from the remodeling observed in the LTCC current, all the other ionic changes described in Sanchez-Alonso et al. [11] representing DCM cardiomyopathy were also included in the model of ICM. Thus, within the cell model, the formulation of the LTCC current is what differentiated between the ICM and DCM cases.

Finally, electrophysiological differences between epi‑ and endocardial cells in each etiology were represented. As in the previous study [11], the approach by Elshrif et al. [68] was used to represent all ionic remodeling in epi‑ and endocardial cells. Within each of these models, the formulation of the LTCC current was the only difference between ICM and DCM cases.

Using the three models described above (control, DCM and ICM), for both endocardial and epicardial myocytes, single-cell simulations were performed using both a voltage-clamp protocol to analyze the whole-cell LTCC current behavior, and a pacing protocol in order to observe differences in action potentials and the possible emergence of cellular-level triggers of arrhythmias such as EADs.

### 2.7 Whole-heart modeling approach

To evaluate if the EADs observed in the cell-level simulations would develop into reentrant arrhythmias in a human heart, the ionic models for both endocardial and epicardial cardiomyocytes were implemented in an MRI-derived, anatomically realistic, healthy human ventricular model. The same model was utilized in our previous study [11], in which transmural electrophysiological differences (epi‑ vs. endo-) in the ventricles were represented by dividing the ventricular walls into an epi‑ and endocardial layers and using the corresponding ionic model for each region based on experimental data from human left ventricle [69]. A computational mesh with resolution of <300 μm was generated and fiber orientation was assigned using a rule-based approach [70]. Stimuli were applied at the apex of the ventricles using a 2.5 mm virtual electrode. Two initial stimuli were delivered at 1 s intervals, followed by a pause in stimulation representing two skipped heart beats, as done previously [11]. After 4 s from the beginning of the simulation, regular pacing at 1 Hz was resumed. Simulations were performed using the CARP software package [71,72].

#### 2.7.1 Deposited data

Experimental datasheet for the figures is available:

https://data.mendeley.com/datasets/9mbxk5mcpp/draft?a=a77df96a-59e4-4602-8af1-96e649f8a31d

#### 2.7.2 Statistics

All statistical analysis and graphs were performed using GraphPad prism. To test normality a Kolmogorov-Smirnov test was used. To test for statistical differences on normal distribution data, One-Way ANOVA was used. When data failed the normality test, the nonparametric Kruskal-Wallis test was used. All data are expressed as mean ± standard error of the mean (SEM). A value of *P*<0.05 was considered statistically significant. All data and statistical tests used in this work is available in the supplemental datasheet.

## 3 Results

### 3.1 Disruption of cellular structures in human failing cardiomyocytes

**Fig. 1:**
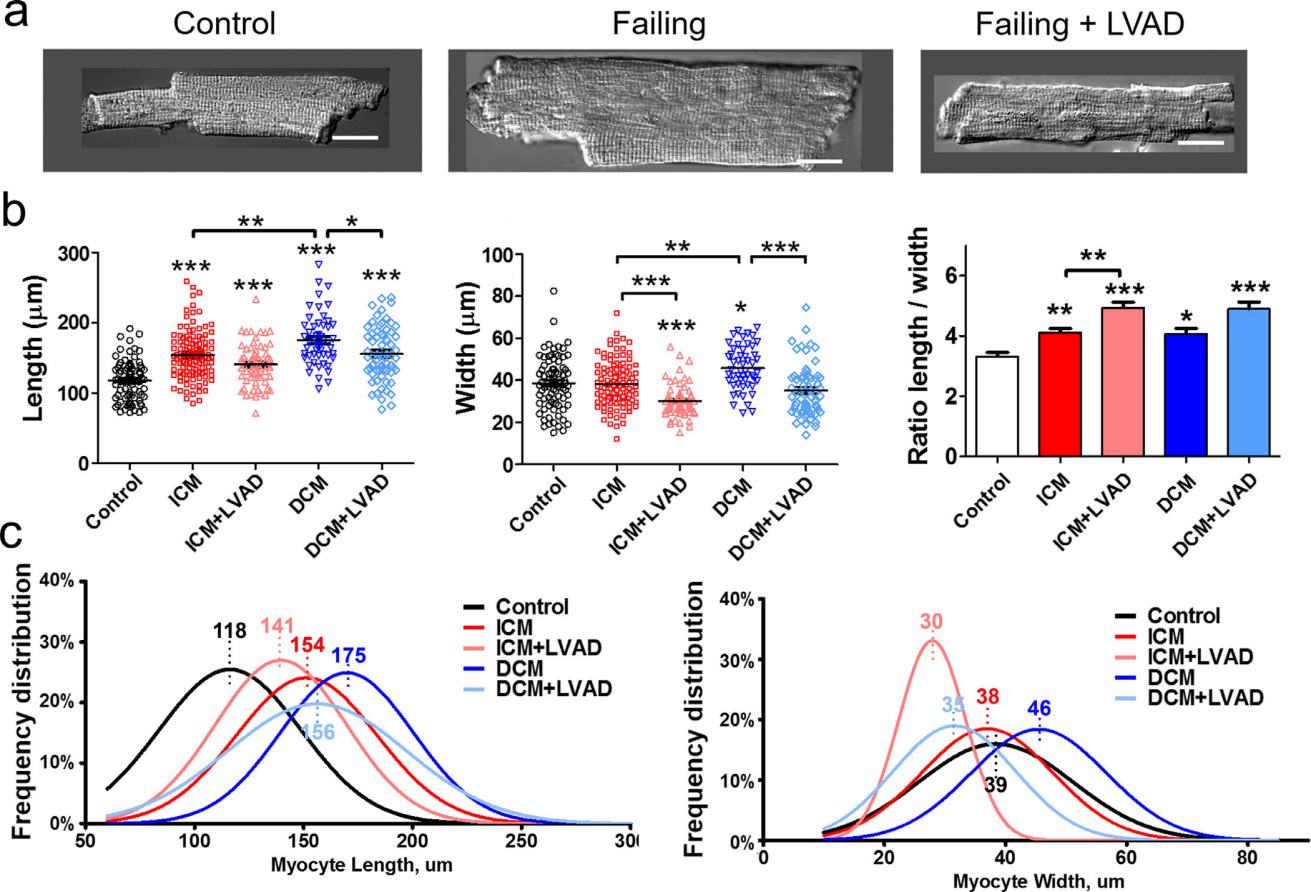
Human ventricular cardiomyocyte size is increased in failing cells and partially reverts after LVAD implantation. **(a)** Representative images of single cardiomyocytes from control, failing and failing with LVAD patients, scale bar 20 µm. **(b)** Length, width and their ratio from control (black, 80 cells), ICM (red, 112 cells), ICM+LVAD (pink, 64 cells), DCM (blue, 52 cells), and DCM+LVAD (light blue, 65 cells) groups. **(c)** Histograms of frequency distribution of individual cardiomyocyte dimensions from b. In each plot, x-axis indicates percent of cells in each size range. Average length and width for each group is represented in the top of each graph. Data are represented as mean ± SEM. * denotes *p*<0.05, ** denotes *p*<0.01, *** denotes *p*<0.001. One-Way ANOVA (length) and Kruskal-Wallis (width and ratio) tests were used.

A well-known characteristic phenotype of human failing cardiomyocytes is their increased size [18,19], which was observed in all the cases studied in this work (Fig. 1); the average cell length increased by 20–49%. Interestingly, DCM cells showed a statistically higher level of hypertrophy than ICM cells (*p*<0.01). LVAD implantation reduced the size of failing cells significantly, as described previously [19], with a remarkable decrease in cell width; the decrease in cell length was less substantial.

**Fig. 2:**
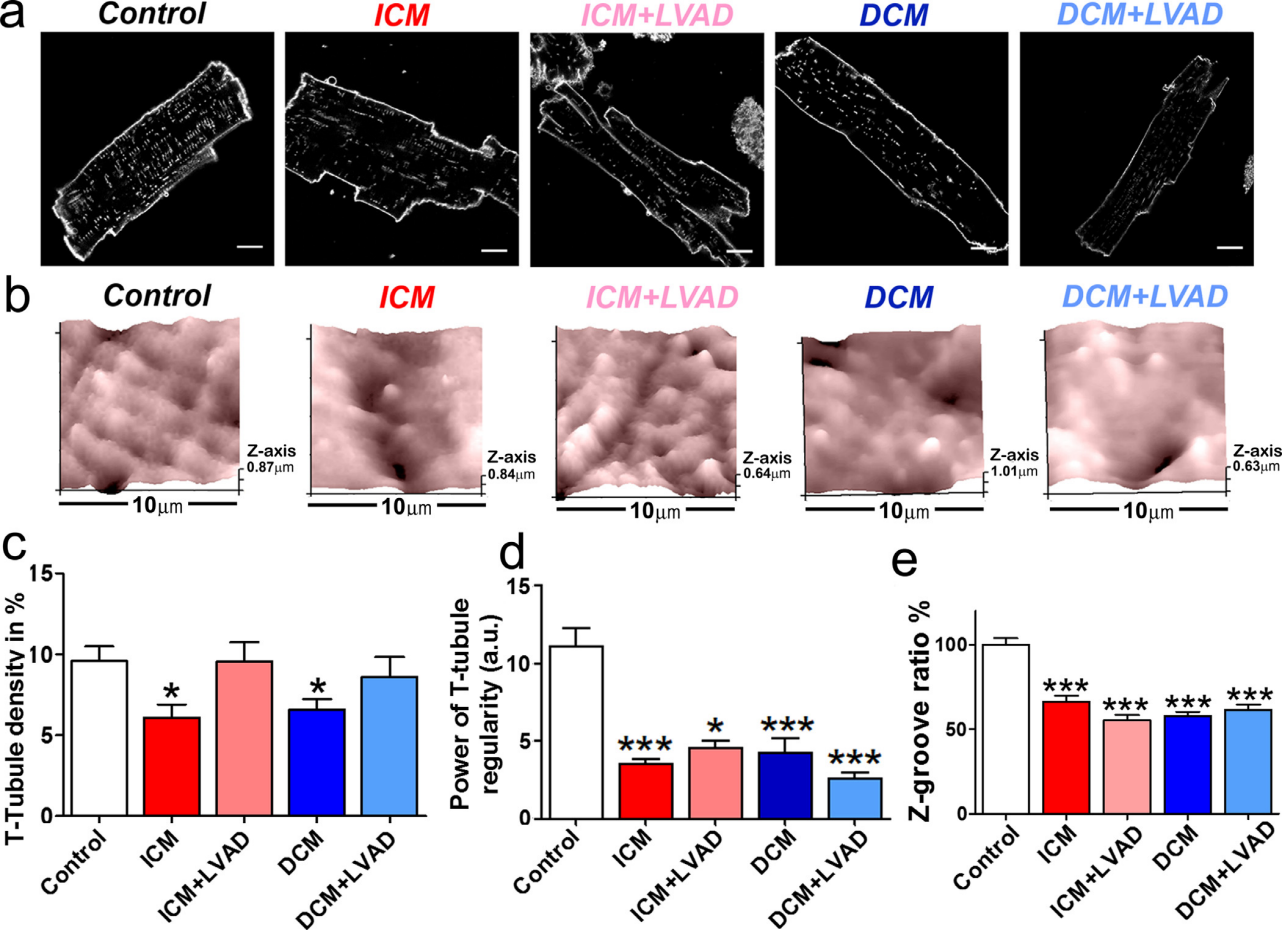
Structural loss in failing ventricular myocytes. **(a)** Confocal image examples of human control and failing cardiomyocytes showing membranes stained with di-8-ANNEPS, scale bar 10 µm. **(b)** SICM 10 µm x 10 µm scans examples from the cell surface shows regular undulations, indicating spatially alternating TT invaginations and surface membrane crests in control and failing cardiomyocytes. **(c)** TT density in control and failing cardiomyocytes (Control *n* = 35, ICM *n* = 28, ICM+LVAD *n* = 14, DCM *n* = 32, DCM+LVAD *n* = 23). **(d)** Power of TT regularity in control and failing cells (Control *n* = 35, ICM *n* = 21, ICM+LVAD *n* = 13, DCM *n* = 32, DCM+LVAD *n* = 23). **(e)** Z-groove index in cardiomyocytes normalized to control average value (Control *n* = 96, ICM *n* = 68, ICM+LVAD *n* = 70, DCM *n* = 116, DCM+LVAD *n* = 80). Data are represented as mean ± SEM. * denotes *p*<0.05, *** denotes *p*<0.001. One-Way ANOVA (TT density) and Kruskal-Wallis (TT regularity and Z-groove ratio) tests were used.

These changes in cell shape were accompanied by deeper cytostructural changes. In human failing myocytes from both ICM and DCM patients, we observed a significant decrease in regularity and internal density of T-tubules as compared to controls (Fig. 2; decrease of 36.3% for ICM TT density, *p*<0.05; decrease of 31.3% for DCM TT density, *p*<0.05; decrease of 68.6% for ICM TT regularity, *p*<0.001; decrease of 61.77% for DCM TT regularity, *p*<0.001), consistent with our previous findings [11]. Interestingly, LVAD implantation in HF patients improved or maintained the density of TTs but did not help recover TT regularity (Fig. 2a, 2c and d), which suggests a lack of improvement in cytoarchitecture. After imaging the cell surface topography with SICM, we quantified the Z-groove index in all cell groups as a metric of surface integrity [20], using 10 × 10 µm images. A reduction of 27% to 41% was found in all human cardiomyopathy groups (Fig. 2b and 2e). A significant reduction in the number of TT openings on the surface of failing cardiomyocytes was also found, identified as dark circles in SICM images (Control: 6.5 ± 0.3, *n* = 96, versus ICM: 5.3 ± 0.3, *n* = 68, *p*<0.05; ICM+LVAD: 4 ± 0.2, *n* = 70, *p*<0.001; DCM: 4 ± 0.2, *n* = 116, *p*<0.001; DCM+LVAD: 4.4 ± 0.2, *n* = 80, *p*<0.001; Supp. Figure S1).

### 3.2 Redistribution of L-type calcium channels on the membrane of human failing cardiomyocytes

**Fig. 3:**
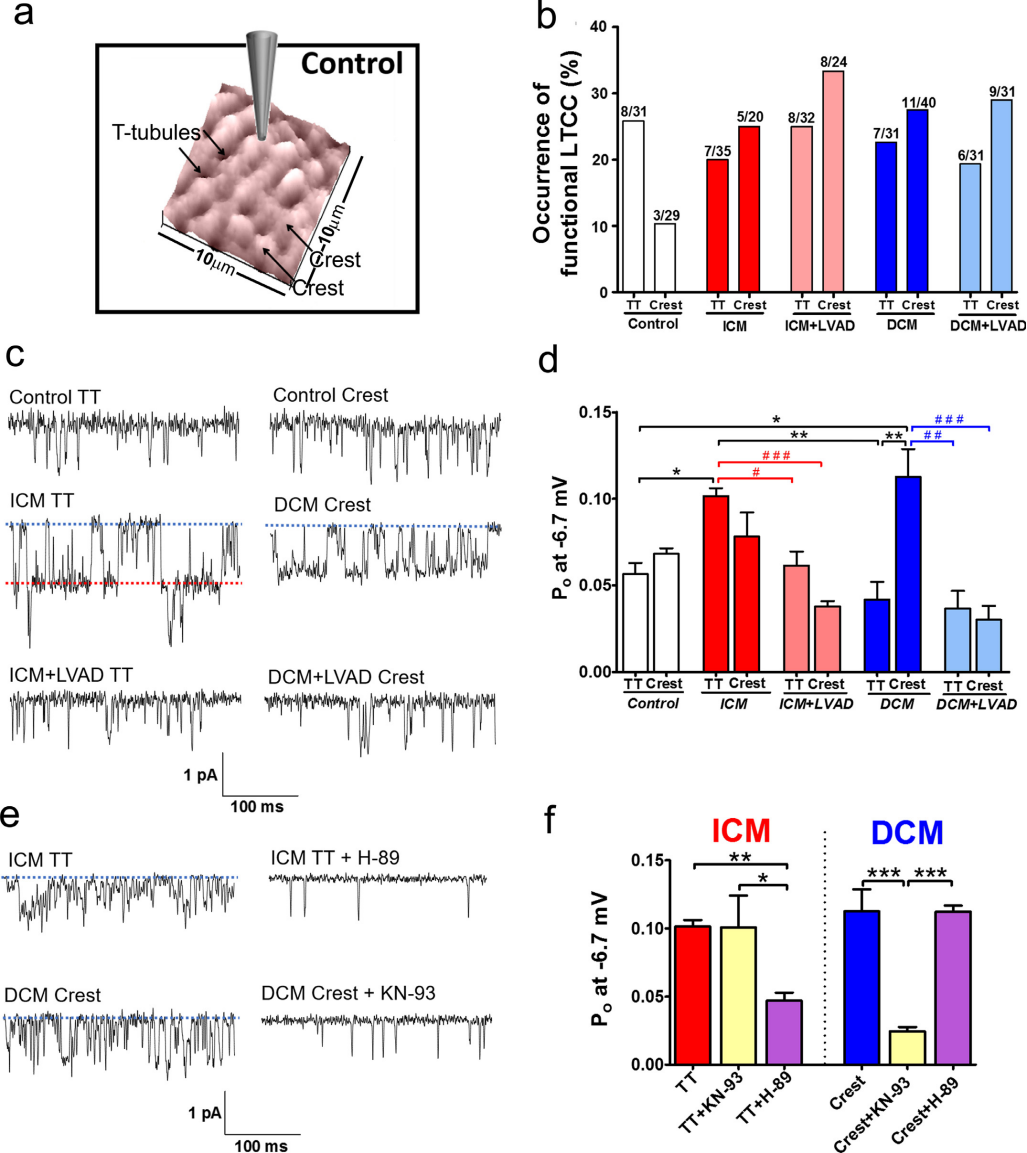
LTCC localization and characteristics in control versus failing ventricular cardiomyocytes. **(a)** 10 × 10 µm representative SICM topographical image of a control cell showing the location of TT and Crest microdomains. **(b)** Representation of the chance of obtaining a LTCC current (% of occurrence). It represents the number of recordings with LTCC activity (left number in the bar) versus the total number of recordings done in a specific microdomain and group (right number in the bar). **(c)** Representative single channel traces at −6.7 mV. **(d)** Graph showing the P_o_ of TT or Crest channels in all the groups. P_o_ is increased on TT of ICM cells, and on Crest of DCM cells. LVAD implantation recover these values to control levels (n number TT/crest: Control 13/11; ICM 9/8; ICM+LVAD 15/13; DCM 11/13; DCM+LVAD 9/13). **(e)** Representative single channel traces at −6.7 mV with or without kinase blocker. **(f)** Graph showing how the P_o_ of ICM and DCM channels decrease after the application of H-89 or KN-93 (ICM: TT *n* = 9, TT+KN-93 *n* = 5, TT+*H*-89 *n* = 7, DCM: Crest *n* = 12, Crest+KN-93 *n* = 13, Crest+*H*-89 *n* = 10). Data are represented as mean ± SEM. * denotes *p*<0.05, ** denotes *p*<0.01. Kruskal–Wallis test was used.

It has been shown that the loss of TT and alterations in the surface topology of the cellular membrane are related to the changes of the LTCCs distribution [11,12]. The channels, located predominately in the TT in healthy cells, are redistributed to crest areas in failing cells, where normally they are rarely recorded. Confirming this result, an increase in LTCC occurrence on the crest was found in all HF samples, from 10.3% of crest patches showing LTCC activity in control cells to 25–33.3% in failing cells (Fig. 3a and 3b). On the TTs, a slight decrease in occurrence was observed in failing cells (19.3–25%) as compared to controls (25.8%). LVAD implantation was found to not recover the normal distribution of LTCCs, keeping the imbalance of more channels recorded on the crest than in the TT microdomain.

An estimation of active channels density in each microdomain was also done (see Table 1 and related Method of calculation). A higher LTCC density was found in TT (3.94 channels/µm^2^) versus crest (2.14 channels/µm^2^) in control cells, with a ratio of 1.84. However, in failing cells, this ratio is reduced (ICM ratio: 0.68, ICM+LVAD ratio: 1.11, DCM ratio: 1.46, DCM+LVAD ratio: 1.45), which can be attributed to the loss of channel density in TT in failing cells, suggesting that the loss of TT structure in HF is also linked to a loss of active LTCCs in the remaining structurally-intact TTs.

**Table 1:** Summarize of cell-attached LTCC recordings. The total number of channels is divided by the total area sealed to obtain an estimation of the LTCC density in the surface of cardiomyocytes. Bay K 8644 group represents control cardiomyocytes recorded under the LTCC agonist stimulation. Myo+LVAD represent the recordings from the Myocarditis + LVAD heart sample.

Group		Total cells sealed	Occurrence (seals that show LTCC activity)	Total area sealed (µm^2^)	Total number of channels	Average channels per seal with LTCC	Estimated Density (channel/ µm^2^)
*Control*	***TT***	31	25.8%	3.30	13	1.63	**3.94**
	***Crest***	29	10.3%	5.14	11	3.66	**2.14**
*ICM*	***TT***	35	20%	4.92	9	1.29	**1.83**
	***Crest***	20	25%	3.34	9	1.8	**2.7**
*ICM+LVAD*	***TT***	32	25%	3.75	12	1.5	**3.20**
	***Crest***	24	33.3%	3.83	11	1.37	**2.87**
*DCM*	***TT***	31	22.6%	2.93	10	1.43	**3.42**
	***Crest***	40	27.5%	6.39	15	1.36	**2.35**
*DCM+LVAD*	***TT***	31	19.3%	3.21	9	1.5	**2.8**
	***Crest***	31	29%	6.75	13	1.44	**1.93**
*Bay K 8644*	***TT***	13	30.7%	1.58	16	4	**10.11**
	***Crest***	16	18.8%	2.58	13	4.3	**5.05**
*Myo+LVAD*	***TT***	14	21.4%	2.44	4	1	**1.64**
	***Crest***	13	23.1%	1.36	3	1.33	**2.21**

**Table 2:** Clinical characteristics of heart failure patients. Values are total numbers of patients with percentages in parentheses unless indicated otherwise. ACE, Angiotensin-converting enzyme inhibitors; ARB, Angiotensin II receptor blocker.

Characteristics				
Diagnosis	DCM	DCM+LVAD	ICM	ICM+LVAD
Total number of patients	16	8	6	4
Age, year (mean ± SD)	49±9	46±6	57±7	52±13
Men, n (%)	12 (75)	5 (63)	6 (100)	4 (100)
Women, n (%)	4 (25)	3 (37)	0 (0)	0 (0)
Surgical procedure				
Coronary artery bypass surgery	0 (0)	1 (13)	5 (83)	3 (75)
Coronary artery bypass grafting	0 (0)	0 (0)	2 (33)	0 (0)
Percutaneous coronary intervention	0 (0)	1 (13)	5 (83)	3 (75)
Medical history				
Previous myocardial Infarction	0 (0)	1 (13)	5 (83)	3 (75)
Diabetes mellitus	1 (6)	1 (13)	1 (17)	0 (0)
Hypertension	2 (13)	1 (13)	1 (17)	0 (0)
Ex-smoker	9 (50)	2 (25)	4 (67)	4 (100)
Alcohol (more than 5 units per week)	4 (25)	3 (38)	1 (17)	2 (50)
Medications				
Antiplatelets	12 (75)	8 (100)	6 (100)	4 (100)
Diuretics	16 (100)	5 (63)	5 (83)	4 (100)
Aldosterone antagonist	9 (56)	8 (100)	3 (50)	3 (75)
β-blockers	14 (88)	6 (75)	5 (83)	3 (75)
Statins	4 (25)	0 (0)	5 (83)	3 (75)
Ca^2+^ channel blockers	0 (0)	0 (0)	0 (0)	0 (0)
Antiarrhythmics	6 (38)	5 (63)	1 (17)	2 (50)
ACE and ARB	11 (69)	6 (75)	5 (83)	4 (100)

LTCC agonist Bay K 8644 was used on control cells to record the maximum density of channels available in the surface of the cardiomyocytes (Table 1 and Supp. Figure S2). Under stimulation, channel density was dramatically increased in both microdomains (10.11 channels/µm^2^ in TT and 5.05 channels/µm^2^ in crest) confirming that in TT the density of LTCCs is higher than in crest (Control ratio TT/Crest: 1.84 channels/µm^2^, vs Bay K 8644 ratio TT/crest: 2 channels/µm^2^).

Surprisingly, the higher chance of finding LTCCs on the crest of failing cells (control occurrence 10.3% vs failing 28.7%) was not found to be linked to a higher density of LTCCs (control: 2.14 channels/µm vs failing: 2.36 channels/µm^2^). In fact, observed clusters of functional channels in the crest of control cells were lost in failing cells. The 3 recordings showing LTCC activity in the control crest microdomain present multiple channels (11 channels in total, 3.66 average channel per seal). In contrast, in failing cells, from 32 recordings showing LTCC activity in the crest, only 12 present multiple channels (48 channels in total, 1.45 average channel per seal).

### 3.3 LTCC activity is increased in specific microdomains in human failing cardiomyocytes

Single LTCC activity from control and failing human cardiomyocytes was elicited by 1 s depolarizing pulses from a holding potential of −96.7 mV to test potentials between −36.7 to 23.3 mV (Supp. Figure S3a, b). The average amplitude from all the openings during the pulse was plotted against the test potential for every experiment, to determine the conductance as the mean of the slopes of this linear relationship. No significant statistical differences were found between TT and crest LTCCs in control cells (TT: 13.74±1.13 pS vs crest: 16.05±1.08 pS, *p*>0.05) or in any of the failing groups (Supp. Figure S3c, d). The low conductance reported here may be lower than expected due to amplitude underestimation, because of lack of fully resolved openings for some of the voltages studied. To facilitate full openings at all test potentials, the LTCC agonist Bay K 8644 (5uM) was applied in the pipette in a sub-set of experiments in control cells. Under stimulation, the slopes of the linear I/V relationships were 24.94±0.4 for TT LTCCs and 23.72±0.95 for crest LTCCs (*p*<0.001 vs control TT and *p*<0.01 vs control crest), consistent with data from other authors[21].

The P_o_ was determined for all the channels at a voltage of −6.7 mV (Fig. 3c and 3d). LTCC P_o_ was significantly increased in the crest of failing DCM myocytes as compared to that in the TT of control myocytes (P_o_: 0.057±0.0065, *n* = 13 for control TT vs. 0.113±0.0161, *n* = 13, for failing DCM crest, *p*<0.05), without changes in P_o_ of the DCM TT LTCCs, as it has been recently shown [11]. Interestingly, for ICM cardiomyocytes, TT channels were associated with elevated P_o_ (P_o_: 0.057±0.0065, *n* = 13, for control TT vs. 0.102±0.0046, *n* = 9, for failing ICM TT, *p*<0.05), without a statistically significant increase in P_o_ of the crest channels, indicating a different pathological substrate between DCM and ICM patients.

Deployment of LVAD in patients results in a reduction of P_o_ for all the channels analyzed. Specifically, the channels found pathologically active in DCM patients in the crest and in ICM patients in the TT showed a significant reduction in P_o_ following LVAD implantation (P_o_: 0.03±0.079, *n* = 13, for DCM+LVAD crest vs. 0.113±0.0161, *n* = 13, for DCM crest, *p*<0.01; P_o_: 0.062±0.082, *n* = 15, for ICM+LVAD TT vs. 0.102±0.0046, *n* = 9, ICM TT, *p*<0.05, Fig. 3d).

### 3.4 Involvement of PKA and CaMKII pathways in the progression of human HF diseases

Elevated phosphorylation status is a mechanism reported to increase P_o_ of the LTCC [10]. We tested whether LTCC phosphorylation by CaMKII or by PKA was related to the increase of P_o_ in failing human cardiomyocytes.

It has been shown that CaMKII can phosphorylate LTCCs in cardiomyocytes [22] and that CaMKII activity is increased in HF [23]. In previous results from a rat model of HF we showed how elevation of CaMKII is responsible for hyperactive LTCC channels located in the crest [11]. We tested if this result can be confirmed in human cardiomyocytes from failing samples (Fig. 3e and f). As expected, in DCM patients, blockade of CaMKII reduced the Po of LTCC crest channels, from a Po of 0.113±0.0161 on DCM crest to a Po of 0.025±0.0031 on DCM crest LTCCs treated with KN-93 (*p*<0.01). However, in ICM myocytes, blocking CaMKII did not reduce the Po of the hyperactive LTCC located in the TT (from 0.102±0.0046 for ICM TT to 0.101±0.025 for KN-93 treated ICM TT). PKA is also a well-established regulator of LTCC function [24]. Due to the lack of an effect of KN-93 on the ICM TT channels, we tested if PKA could be responsible for the increased LTCC P_o_ (Fig. 3e and 3f). Indeed, blocking PKA with H-89 reduced the P_o_ of the ICM TT channels (0.102±0.0046, *n* = 9, for ICM TT vs. 0.047±0.0057, *n* = 6, H-89 treated ICM TT, *p*<0.01), without affecting DCM crest channels (0.113±0.0161 on DCM crest vs. 0.112±0.0045, *n* = 10, H-89 treated DCM crest). This suggests that two different mechanism are responsible for phenotype observed: in DCM CaMKII in the crest microdomain is pathologically active, whereas in ICM PKA is over-active in the TT domain.

Next, we used computational modeling to provide mechanistic insight into how the sub-cellular microstructure changes in HF can lead to electrical dysfunction at the organ level, and specifically, how the phosphorylation state of LTCCs in different microdomains can trigger arrhythmogenic events in the whole heart.

### 3.5 Cellular level computational modeling predicts abnormal L-type calcium channel behavior

**Fig. 4:**
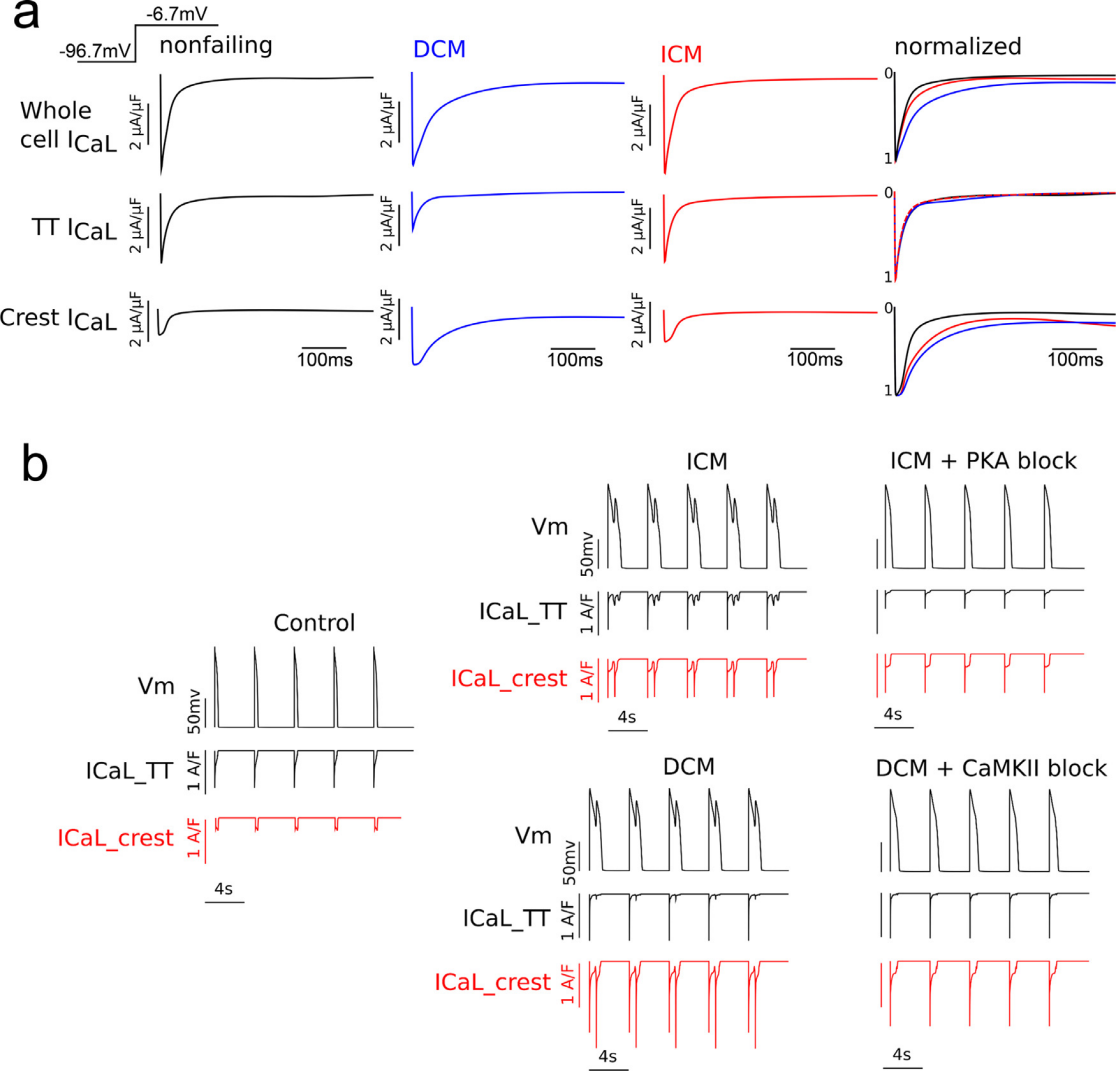
Computational modeling. **(a)** Overview of whole L-type calcium current model under voltage-clamp protocol. LTCC current from the control (black), DCM (blue) and ICM (red) models. Currents were normalized and plotted on the same graph to compare their decay rates. The three rows represent the whole cell LTCC current (first row), the TT component (second row) or the crest component (third row). **(b)** Comparation of membrane voltage and L-type Calcium current traces. For the ICM model, EADs were obtained when the LTCCs in the TTs were phosphorylated by PKA (top row, left), but not when PKA activity was blocked (top row, right). Similarly, arrhythmogenic triggers developed in the DCM model when LTCCs from the crest were phosphorylated by CaMKII (bottom row, left), and not when CaMKII activity was blocked (bottom row, right).

In single-cell voltage clamp simulations using either the endocardial or epicardial myocyte models described in the Methods section, stepping the membrane voltage from −96.7 mV to −6.7 mV, as in the experimental protocol here, led to an influx of Ca^2+^ ions into the cell similar in magnitude across all the three cases (control, DCM and ICM, Fig. 4), which agrees with previous findings [7], [8], [9]. However, the rate at which these currents decayed is different, with the control case displaying the fastest rate, and the DCM being the slowest. This suggests that LTCC current could be a potential cause of action potential prolongation. Examining the TT I_Ca,L_ component, we found that only in DCM myocytes the I_Ca,L_ current was significantly smaller. Thus, although there are fewer channels in the TT domain in both cardiomyopathy aetiologies, the 2.5 increase of the TT current by PKA-phosphorylation compensated for the loss of channels in ICM myocytes. In terms of current decay rates, all groups showed similar values of the TT component of I_Ca,L_, even ICM myocytes, despite PKA phosphorylation and higher Po. This result can be explained by a calcium dependent inactivation mechanism within the dyadic space. In the TT, the high Ca^2+^ concentration rapidly inactivates the channels in all cases and keeps a close control of channel opening, thus leading to currents with very similar decay rates. On the other hand, in the crest microdomain the calcium dependent inactivation is lower; since calcium does not accumulate as much, voltage dependent inactivation dominates, and thus all channels there display a slower decaying current than in TT. This slower decay of I_Ca,L_ is exacerbated in the DCM case by the phosphorylating effect of CaMKII on crest LTCCs, leading to a higher-amplitude and a slower-decaying current.

These changes in LTCC kinetics (Fig. 4a) led to emergence of EADs at the cellular level (Fig. 4b). Blocking the phosphorylating effects of PKA and CaMKII on LTCCs in the pathological cases led to the disappearance of EADs under an identical protocol (Fig. 4b), which indicates that the two molecules play key roles in the development of cellular-level triggers of arrhythmia in cardiomyopathies.

To better predict the arrhythmogenic consequence of pathological LTCC activity and relocation, we further conducted whole-heart simulations, exploring the potential emergence of reentrant arrhythmias at the organ level.

### 3.6 Organ-scale simulations predict the development of arrhythmias in DCM, but not in ICM model

**Fig. 5:**
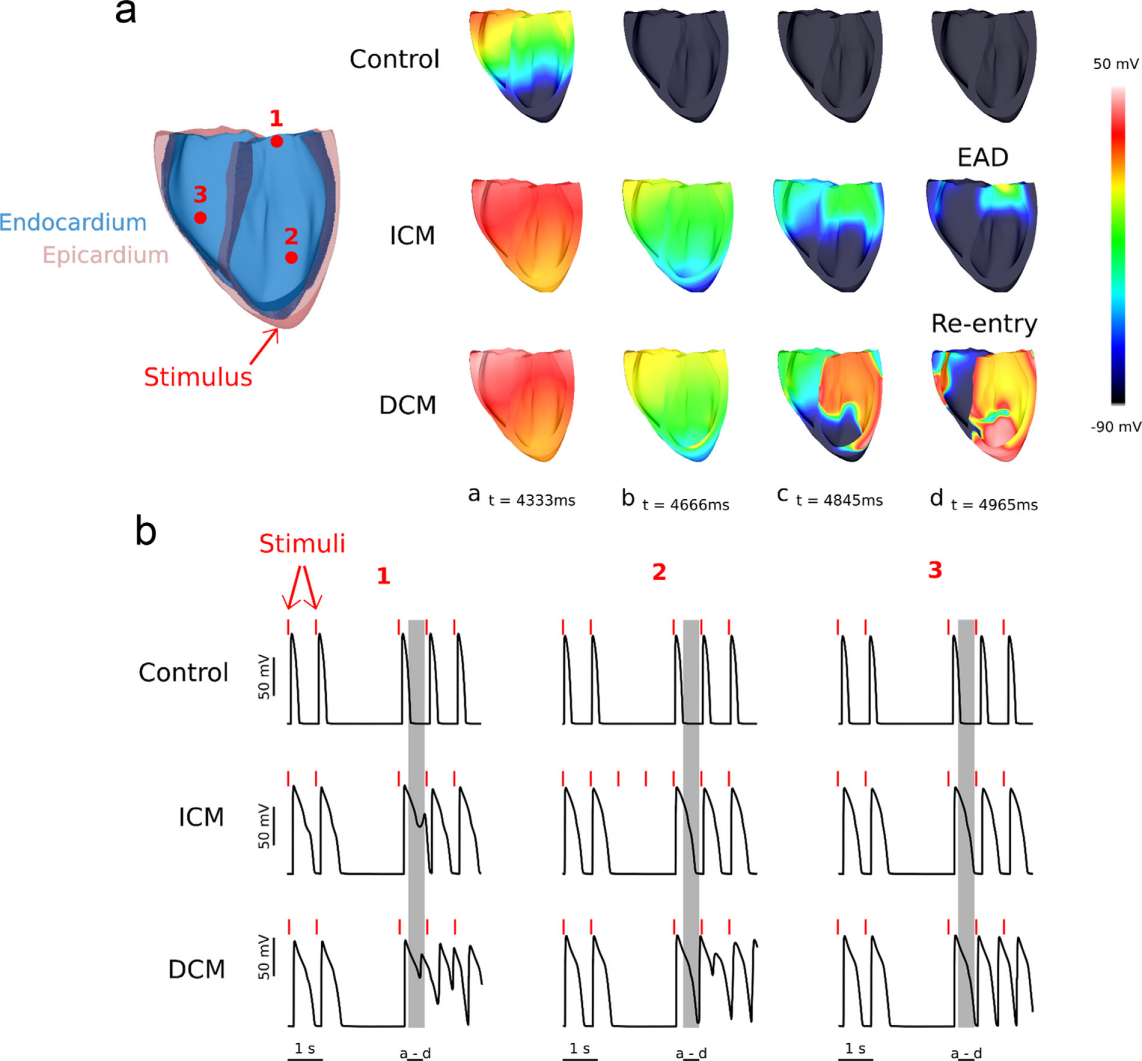
Graphical representation of the whole heart simulations. **(a)** The voltage maps from 4 different points in time were plotted for the three cases (Control, ICM, DCM). A short depolarization between two consecutive stimuli was recorded in the ICM heart, while a reentrant arrhythmia was obtained in the DCM organ. **(b)** The membrane potential was recorded from three different virtual electrodes in the ventricles and displayed. In the ICM case, an island of tissue displayed a synchronous single EAD following the stimulus after the skipped 2 beats. In the DCM case, both singular and multiple EADs were recorded in one or more beats after the skipped ones.

We incorporated in our whole-heart model of human HF published previously [11] the experimentally based ionic model representation for each disease. As a consequence of the presence of EADs in the single cells, arrhythmogenic activity was observed at the whole-heart level. The formation of arrhythmogenic triggers only in the ICM ventricles and of both arrhythmogenic triggers and reentrant arrhythmia in the DCM ventricles is illustrated in Fig. 5 and in Supplementary Videos 2 and 3. No arrhythmic events were observed in the control case (Supplementary Video 1). Action potential duration of the first two beats following stimulation was found to be significantly increased (compared to control) in both pathological cases. Skipping the subsequent two beats to facilitate EAD emergence, as done in previous work at cellular level [11], had no effect in the control ventricles but led to reentrant arrhythmia in the DCM heart model. In contrast, when we skipped two beats in the ICM ventricular model, we observed emergence of EADs only in a small island of tissue, and it did not develop into a reentrant arrhythmia.

Overall, our whole-heart simulations demonstrated that subtle differences in microdomain remodeling between ICM and DCM cardiomyopathies could lead to very different electrophysiological outcomes at the whole-heart level. Specifically, the pathway through which LTCCs become hyperphosphorylated (PKA vs. CaMKII) and the location of these phosphorylated channels (TT vs. crest) causes the emergence of arrhythmogenic triggers in both cases but leads to reentrant arrhythmia only in DCM.

## 4 Discussion

**Fig. 6:**
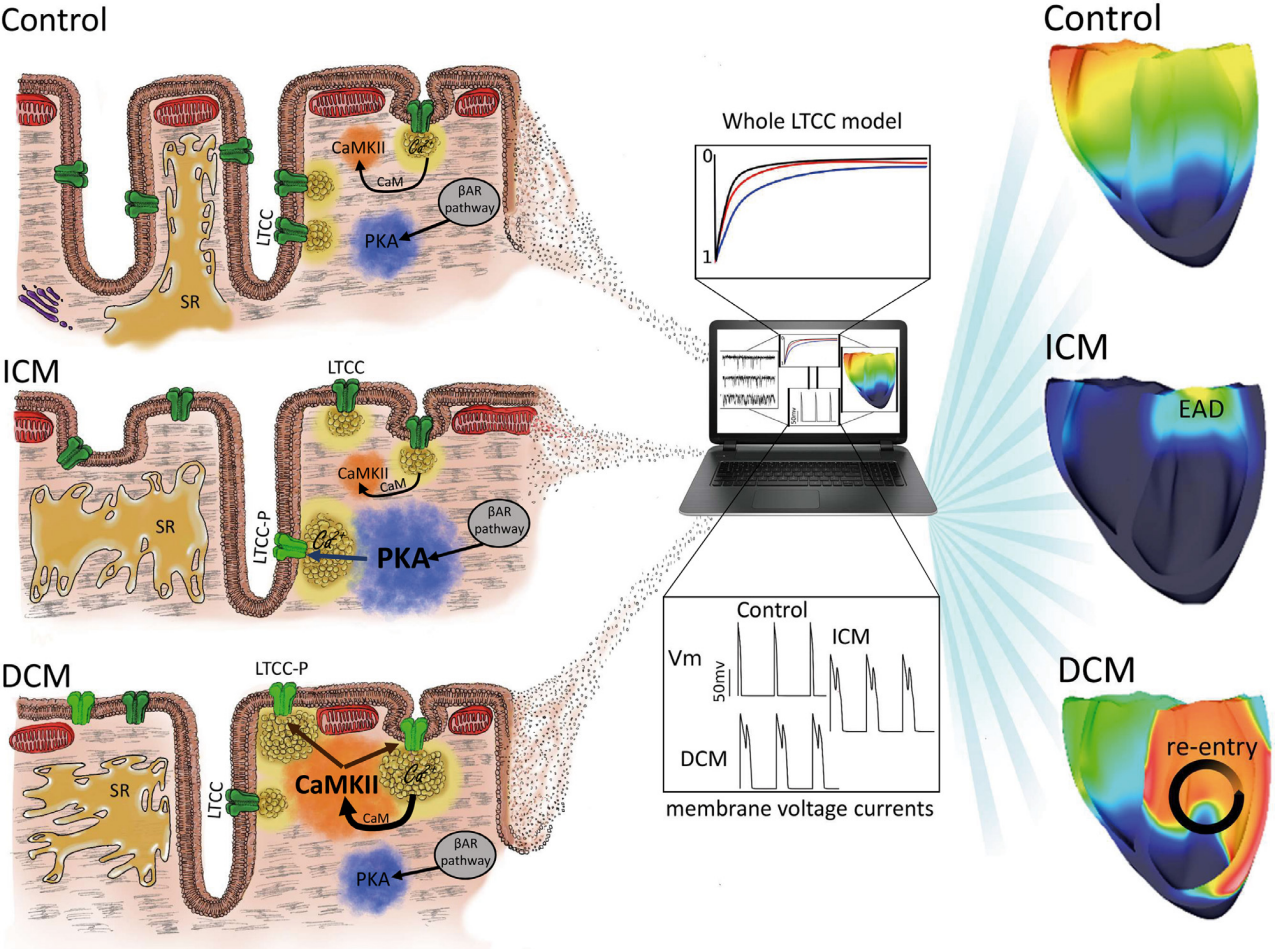
Schematic representation of the mechanism suggested in the differences between ICM and DCM during the progression of HF. A redistribution of L-type calcium channels from the loss of TT to the sarcolemma surface happens in failing cardiomyocytes. At the same time, LTCC channels are phosphorylated (LTCC-P) in the TT of ICM cells, and in the Crest of DCM cells, by an increase of PKA and CaMKII activity respectively. LVAD implantation would produce a decrease of these enzymatic activity leading to a decrease in the open probability of the LTCC. The experimental data was used in a 3D anatomically realistic human ventricular model, showing how LTCC location and activity can trigger pathological events of different severity. BAR: βeta-adrenergic receptor; CaM: Calmodulin; EAD: Early after depolarization.

Altered calcium signaling contributes to the pathology of human HF, and thus its signaling pathways are typically targeted in current therapies for HF [25]. Here, studying specific cellular microdomains using super-resolution scanning patch-clamp [12], we determined how, during human HF progression, the disease etiology determines the spatial location and function of LTCCs. We also show that unloading the heart through LVAD implantation normalizes some of the differences observed between ICM and DCM patients, which suggest that electrophysiological remodeling is a plastic process that can be partially controlled. Finally, combining experimental data with computational modeling enabled us to obtain new mechanistic insights into the emergence of arrhythmogenic triggers and re-entrant arrhythmia. A schematic summary is represented in Fig. 6.

### 4.1 The regression of cellular hypertrophy by LVAD implantation is not associated with restoration of TT structures or the balance of LTCC distribution

Cell hypertrophy could be regarded as a compensatory mechanism during the progression of HF, but it may further impair cardiac performance in the long term. A number of studies have shown that isolated cardiomyocytes from HF patients are larger compared to healthy cells [19,26,27], with similar findings reported in animal models of HF [14]. We confirm both ICM and DCM lead to an increase in cell size in isolated cardiomyocytes, however after implanting LVAD to these patients, a large reduction in the width of the cells is noticeable after isolation (Fig. 1). Interestingly, ICM isolated myocytes, although bigger than the control ones, were smaller in size than DCM cells.

It is well known that the cardiomyocyte TT system is remodeled during HF progression [14], [15], [16]. In cardiomyocytes from final stages of HF we found a reduction of TT density and an impairment of the regularity of these structures along the cell (Fig. 2). This can be partially attenuated by the implantation of LVAD. The TT system in cardiomyocytes from these patients shows a value similar to control samples in terms of TT density, although these TTs are still not as regular as in normal cells. Surface topography (Z-groove index) and the number of TT openings in the surface of the cells were also reduced in all HF groups, including cells from patients with LVAD. This suggests little benefit from LVAD implantation at the level of single cell cytoarchitecture. However, our conclusion about this is biased by our studied group, as only cells from end-stage HF patients in need of heart transplantation were used in this work. In these cases, LVAD does not seem to improve substantially the cell parameters analyzed here, despite the immense benefit that it confers to patients as a bridge to recovery. Interestingly it has been showed that patients with a low degree of TT remodeling by the time of LVAD implantation will achieve high ejection fraction (from 20.7 pre-LVAD to 37.8 post-LVAD), which implies that mechanical unloading in advanced human HF with high degree of TT remodeling does not lead to a recovery of the failing heart [28].

Many studies have found an extensive loss of TT structure in HF [13], [14], [15], [16]. Furthermore, we have uncovered how this loss of structural components leads to an abnormal distribution of LTCCs [11]. In healthy cardiac myocytes, Ca_V_1.2 (the alpha-subunits of the LTCCs) are mainly concentrated in the TT membrane [29]; 3 to 9 times more channels are estimated to be in the TT than in the Crest [30]. This distribution is altered in HF, with channels found to be located more often on the crest of the sarcolemma [11]. In this work, we show that this imbalance in LTCC distribution is a general characteristic of human HF (Fig. 3b and Supp. Figure S4), independent of disease etiology, and that this imbalance cannot be recovered by the implantation of a LVAD.

Taking all together, we conclude that failing myocytes hypertrophy leads to remodeling of the TT system, which manifests in both reduced TT density and fewer TT opening on the sarcolemma, which in turn causes an imbalance in LTCC distribution. Interestingly, LVAD implantation can significantly reduce the size of the cells but is unable to fully recover the TT structures and the balance of the LTCC distribution.

### 4.2 Pro-arrhythmic LTCCs can be attenuated by LVAD implantation

Here, for the first time, we provide a detailed study of single LTCCs activity in different human pathologies, and how the implantation of an LVAD affects it. We found a presence of LTCCs with an abnormally high P_o_ in failing hearts (Fig. 3). These abnormal LTCCs represent a potential pro-arrhythmic substrate, as they lead to AP prolongation [31]. The hyperactivated LTCCs are located in the crest of DCM myocytes and in the TT of ICM myocytes (Fig. 3d). This difference in location could be caused by a different activation pathway during the progression of HF in ICM compared to DCM, in concordance with the differences described previously. It has been suggested that a fraction of LTCCs is phosphorylated under basal conditions in failing human myocytes [9]. Having observed such difference between pathologies, it is logical to suggest that the two phosphorylation pathways act at different levels, one of them targeting the LTCC located in the crest and the other one showing preference for LTCCs in the TT.

Regardless of the pathway involved, it is clear from this work that LVAD implantation causes a significant reduction of LTCC activity in all HF aetiologies (Fig. 3). This may be caused by a global reduction in phosphorylation levels, as it has been suggested to happen in LVAD-supported hearts [9]. The way LVAD implantation reduces the Po of the channels could be a passive process, as it has been shown previously that unloading a healthy heart also reduces the Po of LTCC [32]. Furthermore, we demonstrate that LVAD implantation is associated with a reduction in arrhythmogenic propensity, as blockade of CaMKII and PKA in the simulations resulted in suppression of the EADs in HF cells (Fig. 4).

### 4.3 The etiology of the disease could determine the microdomain-dependent phosphorylation of LTCC, either by PKA or by CaMKII

PKA and CaMKII are two of the main kinases known to modulate LTCC (alpha subunit, Ca_V_1.2) in cardiomyocytes [33]. The activity of the calcium channels is regulated by the interactions of both kinases with the C-terminal part of the alpha subunit of the channel [34,35] and it has been shown that the distal C-terminus of the Cav1.2 is essential for the adrenergic regulation [36]. Altered expression, oxidation, or post-translational changes of both kinases have been associated with the progression of HF [37], [38], [39]. In our previous study we found that the increase of CaMKII activity is responsible for the changes observed in DCM cardiomyocytes [11]. Interestingly, Kirchhefer et al. found that CaMKII activity is increased in human DCM samples, but not in ICM [23]. However, in a more recent study CaMKII was found elevated in both pathologies [40]. Our results show that LTCC activity is increased by CaMKII only in DCM (Fig. 3f). By contrast, in ICM myocytes, LTCC activity is reduced only when PKA activity is blocked (Fig. 3f). However, basal PKA activity was not found to be increased in previous studies of ischemic samples [41]. Even if PKA protein expression is not increased in ICM, post-translational modifications may lead to an increase of PKA activity and a consequent increase of LTCC phosphorylation, as has been shown previously [39].

The β-subunits of LTCC could also play a role in generating the observed differences between DCM and ICM. It has been shown that the overexpression of the β2 subunit leads to the LTCC activity of human cardiomyocytes that mimics chronic heart failure [42]. However, this does not seem to occur through the phosphorylation of PKA sites in the subunit, since the C-terminal sites for PKA are not involved in the adrenergic regulation of the calcium channel [43]. Molecular differences in the Cav1.2 between aetiologies need to be also considered, for example, it has been shown that the Cav1.2 splicing factor Robfox1 is downregulated in DCM, but not in ICM samples [44]. The expression of α and β subunits and the interaction between them in ICM and DCM patients will need to be addressed in future studies.

β_1_ and β_2_ adrenergic receptor (βARs) expression can be also considered to interpret the results. The enhancement of calcium current by βARs could happen through phosphorylation of several residues along the C terminus of the LTCC pore subunit by PKA [45]. However, recently it has been shown that Cav1.2 can be stimulated by βARs without the need of PKA phosphorylating the alpha subunit [46]. It has been proposed that βAR-induced stimulation of Cav1.2 does not need PKA targeting the subunits of the channel, rather an additional protein, Rad is shown to be involved [47]. The specific interaction between β2AR and Cav1.2 has also been unraveled in recent years, from an essential residue in the Cav1.2 for β2AR binding [48], to the activation of different pathways by nano-molar concentrations of β-blockers [49]. CaMKII could also play an important role as a secondary mechanism. Cav1.2 has targets for CaMKII phosphorylation in its structure as well as other regulatory subunits of the channels [50], it is also activated by the βAR pathway [51] and the inhibition of CaMKII on heart failure has been proposed as a therapeutic strategy [40]. Nevertheless, the specific role that β_1_ and β_2_ adrenergic receptors play in the regulation of the LTCC will be the next step for a better understanding of the role of LTCC on human cardiomyopathies.

### 4.4 The outcome of the sub-cellular LTCC distribution on the whole heart is revealed by simulations

Our single-cell simulations showed how the loss of TT and the subsequent redistribution of LTCCs alters cellular action potential. EADs have been widely described in the literature [52], and our simulations demonstrating emergence of EADs in ICM and DCM cells are consistent with these studies (Fig. 5).

In our DCM model, CaMKII phosphorylated LTCCs in the crest, resulting in an altered crest I_CaL_ compared to control, with a higher magnitude and a slower decay (Fig. 4). These changes were the main cause for EAD emergence. In ICM, PKA phosphorylated LTCCs mostly in TT, however, TT I_CaL_ had the magnitude and kinetics of control (Fig. 4), as the loss of LTCCs in this microdomain was compensated by the increase in P_o_ through PKA phosphorylation of the channels remaining in TT. This suggest that the Ca^2+^ handling proteins associated with the TT, such as Na^+^-Ca^2+^ exchanger, sarcolemmal Ca^2+^-ATPase and the SR Ca^2+^ uptake pump [53], are more efficient in TT than on the crest. This reveals new insights into the mechanism of HF, indicating how the location of LTCCs could be more important than their phosphorylation state.

Whole heart simulations demonstrated that LTCC alterations in DCM have worse consequences at the organ level (reentrant arrhythmias) than LTCC alterations in ICM (Fig. 5). This points to the subcellular location as being a key player in the process of LTCCs becoming the trigger of arrhythmogenic events. When LTCCs are located in TT, the machinery of the cell in this microdomain can precisely control calcium influx and even compensate for channel loss with an increase in P_o_. However, when LTCCs are located in the crest, balance is easily disrupted, and an increase in channel number there leads to EADs (as shown by ICM simulations). If this is accompanied by an increase in P_o_ (DCM simulations), electrophysiological disturbances will progress to reentrant arrhythmias.

### 4.5 Study limitations

The number of experiments that could be performed on human cardiomyocytes in different aetiologies is limited by several factors. The widely used inhibitors, H-89 and KN-93, were selected for this project to explore the involvement of PKA and CaMKII in the regulation of LTCC. We cannot disregard the potential involvement of other signaling pathways that could be indirectly affected by these inhibitors. H-89 is described as a blocker of PKC as well, and at higher concentration it had been suggested to block *K*+ currents [54]. In the case of KN-93, there is evidence that it can also binds to the CaM protein, and not only to CaMKII, affecting Nav1.5 or RyR2 function [55]. The conclusions of our study on the involvement of the examined pathways in arrhythmogenic events must be regarded with caution, and more studies should be performed with a wider range of inhibitors.

### 4.6 Clinical implications

The prognosis and survival of patients with HF may benefit from an early diagnosis of the etiology of the disease, thus allowing specific targeted treatment informed by the different sub-cellular pathologies described here. This work presents new insights into the end-stage phenotype of human HF and suggests how different pathologies could result from changes in microdomains at the single cell level. Our modeling approach spanning from stochastic LTCC gating to arrhythmogenesis at the organ level enabled us to understand how subcellular changes can influence the development of arrhythmias. These outcomes may be useful in informing choice of anti-arrhythmic therapies and strategies specific to the patient's pathophysiology and probability of the type of underlying arrhythmic substrate. Historically most calcium-channel blockers in clinical trials had been more detrimental than beneficial in patients with advanced left ventricular dysfunctions [56], [57], [58]. It was showed in the PRAISE study in 1996 that Amlodipine, a calcium channel blocker, could decrease the risk of death by 46% on non-ischemic heart disease patients, without any effect on ischemic patients [59]. However, when the study was extended to a higher population, the favorable effect on non-ischemic patients was no longer observed [60]. This work suggests the importance of phosphorylated LTCC in the development of arrhythmias, mainly on DCM patients, and how the etiology of the disease can determine the subcellular changes. Looking for new treatments based in phosphorylated LTCCs in specific microdomains could improve the prognosis of HF and be the key for calcium channel blockers treatments.

## Declaration of Competing Interest

The authors declare no competing interests.

## Appendix Supplementary materials

Image, video 1

Image, video 2

Image, video 3

Image, application 4
