# Supplementary material for: Nanoscale regulation of L-type calcium channels differentiates between ischemic and dilated cardiomyopathies
Source: eBioMedicine. 2020 Jun 21;57:102845. doi: 10.1016/j.ebiom.2020.102845 (PMC7317229; doi:10.1016/j.ebiom.2020.102845)
Supplement: Supplementary file 4 [file mmc4.docx]

**SUPPLEMENTAL FIGURES**

**
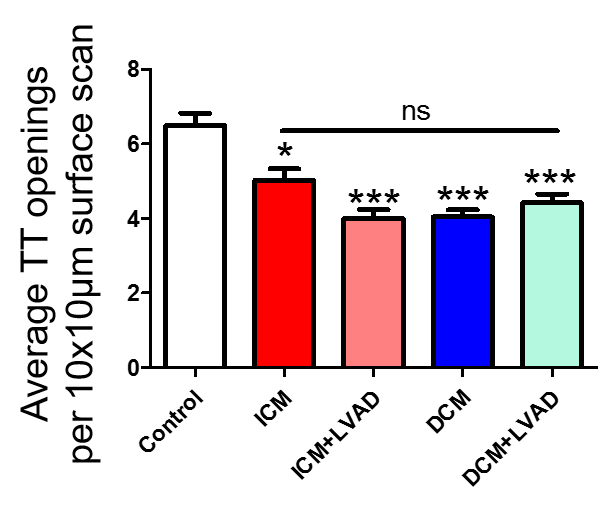
**

**Supplementary Figure S1. Average TT openings in the surface of cardiomyocytes.** The number of T-tubules was counted in a 100 µm2 area in a 10x10µm SICM scan for each cell (control n=96, ICM n=68, ICM+LVAD n=70, DCM n=116, DCM+LVAD n=80). For statistics Kruskal-Wallis test was used. Data are represented as mean ± SEM. * denotes p<0.05, *** denotes p<0.001.


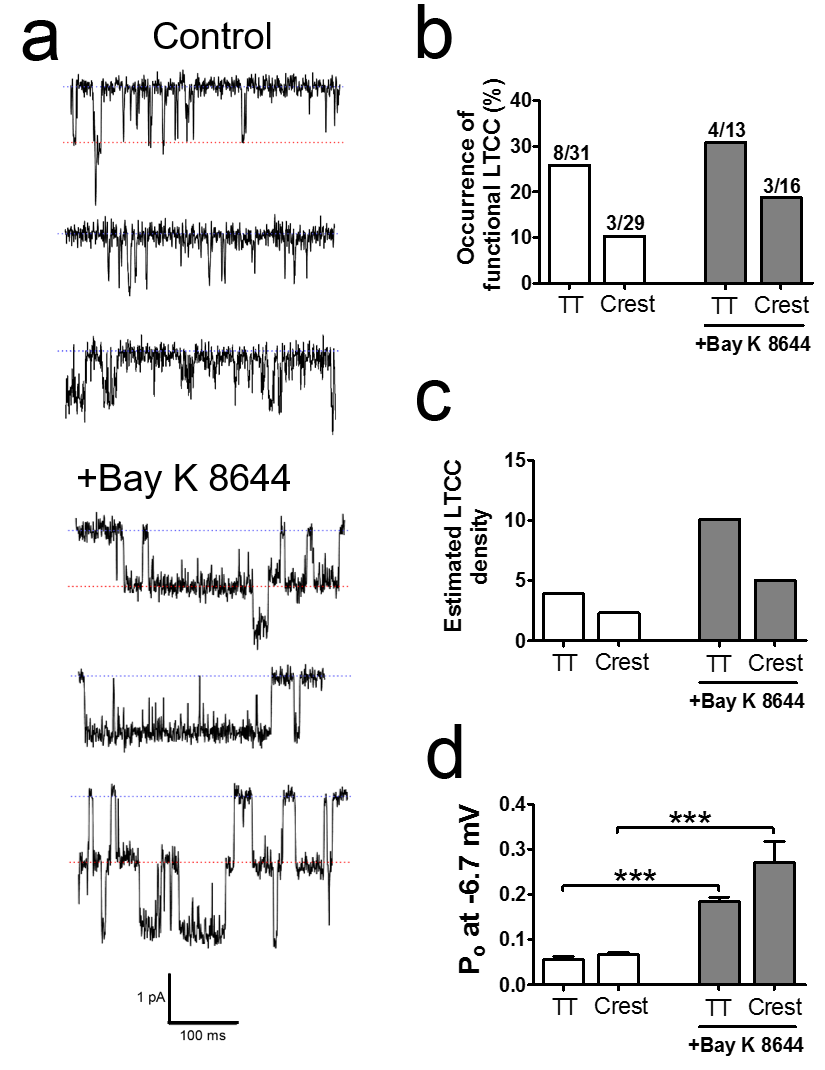


**Supplementary Figure S2. Effect of the LTCC agonist Bay K 8644 on control cardiomyocytes. (a)** Representative single channel traces at -6.7 mV for control (top) and Bay K 8644 treated (bottom) cells **(b)** Graph showing the chance of obtaining a LTCC current (% occurrence) with or without Bay K 8644 (number in the bar indicates recordings with channel / total recordings). **(c)** Estimated functional LTCC density in the surface of cardiomyocytes with or without Bay K 8644 (calculated from LTCC recordings in b). **(d)** Graph showing the open probability of TT or Crest channels. Po is drastically increased in the presence of Bay K 8644 (Control: TT n=13, Crest n=11; Bay K 8644: TT n=16, Crest n=13). Data are represented as mean ± SEM. *** denotes p<0.001


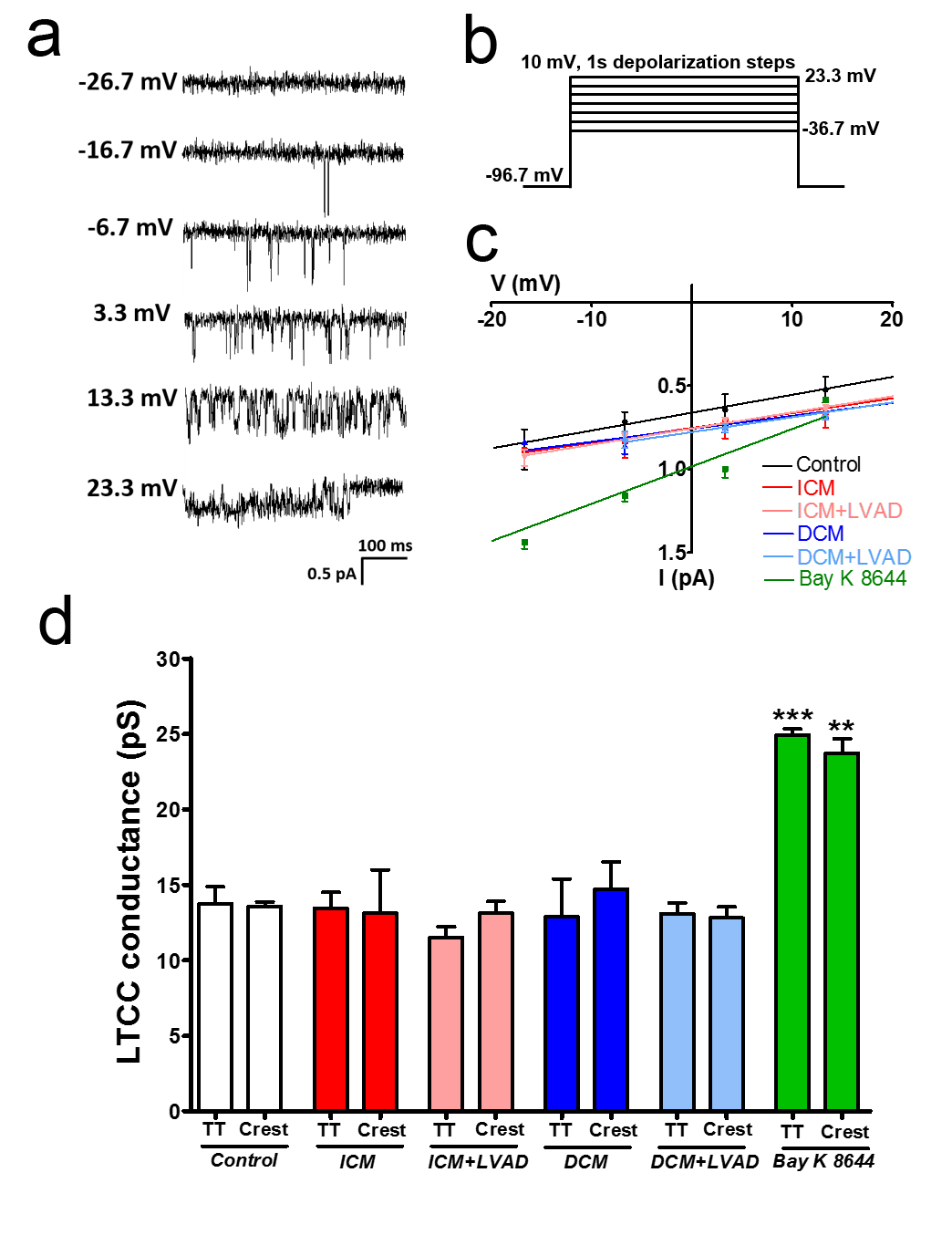


**Supplementary Figure S3. LTCC conductance in control versus failing ventricular cardiomyocytes. (a)** Representative single channel traces at indicated voltages from a control cell. **(b)** LTCC activation protocol, after a seal is made the voltage is held at -96.7mV and 10mV steps of 1s from -36.7 to 23.3 are applied. **(c)** Voltage vs current dependence representation of single LTCC currents of control and failing cardiomyocytes from TT recordings (Control: TT n=10; ICM: TT n=6; ICM+LVAD: TT n=15; DCM: TT n=7; DCM+LVAD: TT n=6; Bay K 8644: TT n=15). **(d)** LTCC conductance was measured for each channel and was unchanged between all groups except the increase observed when control cells where treated with the LTCC agonist Bay K-8644 (Control: TT n=10, Crest n=11; ICM: TT n=6, Crest n=6; ICM+LVAD: TT n=15, Crest n=11; DCM: TT n=7, Crest n=8; DCM+LVAD: TT n=6; Crest n=8; Bay K 8644: TT n=15, Crest n=13). Data are represented as mean ± SEM . ** denotes p<0.01, *** denotes p<0.001.


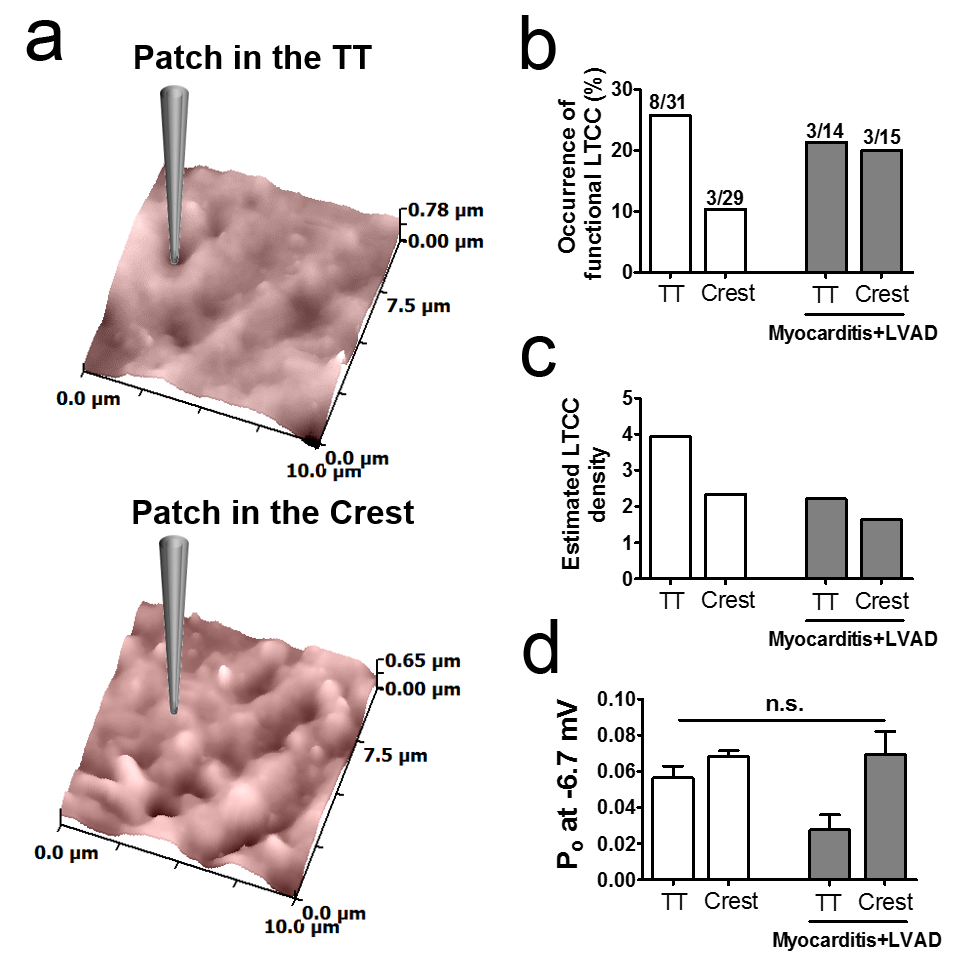


**Supplementary Figure S4. Comparison between cardiomyocytes from control samples and a Myocarditis + LVAD sample. (a)** 10x10µm representative SICM topographical images of disease cells showing the location of a patch in the TT (top) or in the Crest (bottom) microdomain. **(b)** Graph showing the chance of obtaining a LTCC current (% occurrence) in both cases (number in the bar indicates recordings with channel / total recordings). **(c)** Estimated functional LTCC density in the surface of cardiomyocytes of control and Myocarditis+LVAD cells (calculated from LTCC recordings in b). **(d)** Graph showing the open probability of TT or Crest channels. (Control: TT n=13, Crest n=11; Myocarditis+LVAD: TT n=3, Crest n=4). Data are represented as mean ± SEM.

**SUPPLEMENTAL VIDEOS:**

**Online Video 1.**Simulated control ventricular movie showing organized and regular response to each pacing stimulus (1Hz pacing). Two beats were skipped to facilitate EAD emergence. Control simulations showed no changes when the pacing was restarted.

**Online Video 2.**Simulated ICM ventricular movie showing APD prolongation. After the skipped beats (two skipped beats after two pacing stimuli at 1Hz) an EAD can be observed in a small island of tissue but no re-entrant arrhythmia was produced.

**Online Video 3.**Simulated DCM ventricular movie showing APD prolongation. After the skipped beats (two skipped beats after two pacing stimuli at 1Hz) cells failed to repolarize and a re-entrant arrhythmia developed in the whole heart. ​
